# Cortisol Reactivity to a physical stressor in Patients with Depression and Alzheimer's disease

**DOI:** 10.1590/1980-5764-DN-2021-0066

**Published:** 2022

**Authors:** Ana Beserra, Bruno Oliveira, Eduardo Portugal, Patrícia Dutra, Jerson Laks, Andrea Deslandes, Helena Moraes

**Affiliations:** 1 Universidade Federal do Rio de Janeiro, Instituto de Psiquiatria, Rio de Janeiro RJ, Brazil.; 2 Universidade Federal do Rio de Janeiro, Laboratório de Neurociência do Exercício, Rio de Janeiro RJ, Brazil.; 3 Universidade Federal Rural do Rio de Janeiro, Departamento de Educação Física e Esportes, Rio de Janeiro RJ, Brazil.; 4 Universidade Federal do Rio de Janeiro, Laboratório de Fisiologia do Exercício, Rio de Janeiro RJ, Brazil.; 5 Universidade do Estado do Rio de Janeiro, Laboratório de Imunofisiologia do Exercício, Rio de Janeiro RJ, Brazil.; 6 Universidade do Grande Rio, Programa de Biomedicina Translacional, Rio de Janeiro RJ, Brazil.

**Keywords:** Hydrocortisone, Stress, Mechanical, Alzheimer Disease, Exercise, Depression, Hidrocortisona, Estresse Mecânico, Doença de Alzheimer, Exercício Físico, Depressão

## Abstract

Some prevalent mental disorders in the elderly, such as Alzheimer's disease (AD) and major depression disorder (MDD), are associated with chronic stress and consequently with possible dysregulation of hypothalamic–pituitary–adrenal (HPA) axis and cortisol levels in basal conditions or in the reactivity of an acute stressor. However, evidence of cortisol behavior after a physical stressor in patients with AD and MDD is scarce.

**Objective:**

This study aimed to investigate the cortisol reactivity to a single session of physical exercise in patients with MDD and AD and compare it to healthy control (HC) older individuals.

**Methods:**

HC individuals (n=10) and elderly with clinical diagnostic of MDD (n=08) and AD (n=13) were submitted to a single bout of aerobic exercise in a treadmill during 30 minutes of moderate intensity. Salivary cortisol was collected before and after acute stressor. A repeated-measure analysis of variance (ANOVA), spearman correlation, and linear regression were performed.

**Results:**

The repeated-measure ANOVA revealed no interaction for cortisol on the moment×group [F(2.000, 28.000)=1.285; p=0.293] and no effect for group (F=0.323; p=0.727). However, a significant effect for moment [F(1.000, 28.000)=4.930; p=0.035] was found, with a decreased cortisol levels in postexercise for all groups. The effect size (ES) of cortisol reduction was small for patients with MDD (d=0.402) and trivial for patients with AD (d=0.166) and HC group (d=0.090).

**Conclusions:**

All participants show a decreased cortisol reactivity to a physical stressor, which can be associated with an impairment in coping with an acute stressor.

## RESUMO.

A doença de Alzheimer (DA) e o transtorno depressivo maior (TDM) são transtornos que acometem idosos e estão associadas ao estresse crônico e à desregulação do eixo hipotálamo-hipófise-adrenal (HPA), que repercute em alterações nos níveis de cortisol (basal e reatividade).

**Objetivo:**

Investigar a reatividade do cortisol em uma única sessão de exercício físico em pacientes com TDM e com DA e compará-la com a de idosos saudáveis.

**Métodos:**

Indivíduos controle saudáveis (n=10) e idosos com diagnóstico clínico de TDM (n=08) e DA (n=13) foram submetidos a uma única sessão de exercício aeróbio em esteira rolante, durante 30 minutos, em intensidade moderada. O cortisol salivar foi coletado antes e depois do estressor agudo. Na estatística, foram realizadas as análises de variância (ANOVA) de medidas repetidas, correlação de spearman e regressão linear.

**Resultados:**

Não foi encontrada interação para momento x grupo [F (2.000, 28.000)=1.285; p=0,293] e tampouco efeito para o grupo (F=0,323; p=0,727). Todavia, foi observado efeito significativo para o momento [F(1,000, 28,000)=4,930; p=0,035], mostrando diminuição dos níveis de cortisol no pós-exercício para todos os grupos. O tamanho do efeito (TE) foi considerado pequeno para o grupo TDM (d=0,402) e trivial para o DA (d=0,166) e o saudável (d=0,090).

**Conclusões:**

Todos os participantes apresentaram diminuição da reatividade do cortisol a um estressor físico, o que pode estar associado a um comprometimento no enfrentamento de um estressor agudo.

## INTRODUCTION

Life expectancy has increased in several countries in the past decades, impacting on the prevalence of some chronic diseases and even neuropsychiatric disorders related to aging such as major depressive disorder (MDD) and Alzheimer's disease (AD)^1,2^. Although there is a genetic component for their development in late life, some modifiable risks are known^3,4^, such as chronic stress^5^ and aggravation of the disease courses.

Stress system is regulated mainly by hypothalamic–pituitary–adrenal (HPA) axis resulting in cortisol release in the body, which involves the activation of autonomic nervous system and consequently an increase in blood pressure and heart rate (HR), preparing to a life-threatening situation. Moreover, cortisol crosses the blood–brain barrier, achieving some brain regions to promote negative feedback mechanism. High cortisol levels may cause consequent neuron apoptosis impacting in hippocampus atrophy, also observed in patients with MDD^6^ and AD^7^. This mechanism can be aggravated by some other physiological changes related to aging, such as inflammaging, which is a phenomenon with chronic and low-grade inflammation observed in elderly individuals^8^, and wear and tear of chronic exposure to cortisol in the body (allostatic load)^9^.

In this sense, some studies have observed HPA changes in psychiatric disorders, which may result in hypercortisolism or hypocortisolism at rest in older patients with MDD^10,11^ and hypercortisolism in patients with AD^12^. Furthermore, chronic stress can also change HPA reactivity, causing blunted cortisol reactivity and increased cortisol levels after exposure to a stressor agent^13^. A previous meta-analysis with an adult sample observed higher cortisol reactivity in MDD individuals after the psychosocial stress task^14^. In this line, previous studies with older healthy adults have shown a higher cortisol response to pharmacological or psychological challenges compared to younger individuals^15^.

Specifically, to patients with AD, it is widely known to cause a hyperactivity of cortisol in basal conditions and a circadian cortisol hypersecretion in individuals with clinically diagnosis^16^. A recent study demonstrated that higher psychosocial stress reactivity may represent a preclinical sign of cognitive impairment^17^. It can explain some difficulty in these individuals to develop coping strategy to lead with stressor event^18^, mainly those with worsened cognitive performance^19^. Regarding patients with AD, it is also expected a difficulty in responding to a stressful stimulus due to failure to elaborate confrontational and problem-solving actions^19^.

Although previous studies have shown higher cortisol response for patients with MDD and AD, these studies have investigated through psychological stressor tests. Perhaps, few studies in the older sample may be explained by the difficulty to introduce stressor tests for this population. In this sense, another type of stressor agent, such as physical exercise (PE), with a lower cognitive demand during the task, may be more efficient, which promotes already known changes in HPA axis^20^. Most studies have observed higher cortisol levels after a single session of exercise in elderly individuals^21^, notwithstanding it is unknown if MDD and AD older adults show the same response for cortisol.

Thus, this study aimed to investigate the cortisol reactivity to a single session of PE in patients with MDD and AD and compare it to healthy control (HC) individuals. We expected an increase in cortisol hormone after acute PE among groups with more pronounced levels for patients with MDD and AD.

## METHODS

### Protocol and registration

This research is part of a major study entitled “Efficacy of physical exercise in the Treatment of Major Depression, Alzheimer's Disease and Parkinson's Disease” that evaluates the chronic effect of PE. It was approved by the Ethics Committee for Studies on Humans from the Institute of Psychiatry of Universidade Federal do Rio de Janeiro under registration in CAAE: 24904814.0.0000.5263 and was registered under the Brazilian Registry of Clinical Trials (REBEC) protocol RBR-4M3K2C.

### Recruitment and eligibility criteria

We recruited 34 male and female older adults (≥60 years old) with at least 4 years of education attended on Centre for Alzheimer's Disease and Related Disorders in the Institute of Psychiatry at the Universidade Federal do Rio de Janeiro. In this recruitment, three individuals declined to participate in the research, one in MDD and two in HC group. Participants should be diagnosed with MDD and AD according to DSM-IV (Diagnostic and Statistical Manual of Mental Disorders).

All patients were taken medication, and patients with AD were classified into mild and moderate stages according to the Clinical Dementia Rating (CDR). The HC group was recruited through usually familiar patients, neighbors, or colleagues, with age ≥60 years old, at least 4 years of education, no psychiatric disorder, and cognitive decline confirmed by a psychiatric evaluation. The exclusion criteria were severe stage of AD, any other psychiatric disorder, the existence of any musculoskeletal and audiovisual limitation, the use of corticoid medication, and who performed a dental procedure at least 7 days before saliva collection. In all, 10 individuals in HC group, 8 individuals in MDD group, and 13 in AD group were included. Some individuals included in this research were used to workout (walk on treadmill), while others were sedentary. The sample size was calculated using GPower^®^ software version 3.1, and the estimated sample was about 30 individuals.

### Experimental procedure

The experimental procedure was performed in three visits. The first visit took about 2 hours. In this visit, written informed consent, anamnesis (age, education, medication, comorbidities), short-form International Physical Activity Questionnaire (IPAQ) (rating 1–5: sedentary, insufficiently active A and B, active, and very active) involving physical activity over the past 7 days^22^, Global cognitive state assessed by Mini-Mental State Examination (MMSE)^23^, and depressive symptoms by Geriatric Depression Scale (GDS)^24^ were assessed.

In the second visit, 1 week after the first visit, individuals were submitted to a cardiopulmonary effort test in a treadmill ergometer (InbraMed Pro^®^) using a ramp protocol to estimate the maximal oxygen consumption (VO_2max_)^25^. The speed/slope increases every 30 seconds and was defined based on the difference between the initial and final speed. The test was configured to be finished in 10 min and, based on its results, the VO_2max_ was estimated. The electrocardiographic Digital MICROMED^®^ 12-lead was used to monitor and record blood pressure and electrocardiogram traceability to assure no cardiological disorders. Also, weight and height were recorded to determine the body mass index (BMI).

In the third visit, during the mornings, about 1 month after the first visit, the participants carried out a single bout of acute PE on the treadmill. Individuals underwent a 30-minutes walk on a treadmill, including 5 min of warm-up, 20 minutes of moderate intensity (70%VO_2max_), and 5 min of cool down. Blood pressure was recorded in preintervention and postintervention. The subjective rating perceived exertion (RPE) was measured by Borg's scale^26^, with rating ranging from 6 (no exertion at all) to 20 (maximal exertion). HR was obtained by a monitor, recording prestressor, post-stressor, and every 5 min during the acute PE.

### Cortisol assay and collection

All participants were instructed to avoid eating and drinking 1 h before the saliva collection. Salivary cortisol samples were collected in two moments, namely, preexercise and immediately postexercise, which were performed between 11 a.m. and 1 p.m. Before collection, participants were asked about sleep hours in the last night and were instructed about how to collect stimulated saliva. The secretion is promoted by mechanical movements in jaw after rinsing some water in the mouth. After collection, falcon tubes were stored and kept frozen (-20°C) until the cortisol assay.

After defrosted and centrifuged tubes at 3000 RPM for 10 min, the ELISA test (Enzyme Linked Immunosorbent Assay), using a previously validated and commercially available kit (IBL^®^) was performed. The cortisol limit of detection was 20.0 μL, and the intra- and inter-assay variability were considered 2.1 and 5.2%, respectively (within a 20.0–800.0 ng/mL dosage range).

### Statistical analysis

Shapiro-Wilk and Levene's tests were used to evaluate normality and homoscedasticity, respectively, for all data. One-way analysis of variance (ANOVA) (F) was performed to compare groups in terms of age, education, BMI, VO_2max_, MET_max_, sleep hours, HR pre, HR 10 min, HR 15 min, HR 20 min, delta HR (postexercise − preexercise), delta cortisol (postexercise − preexercise), delta RPE (postexercise − preexercise), speed, and slope during the acute physical stressor. Kruskal-Wallis (χ^2^) was calculated for MMSE, GDS, IPAQ classification, IPAQ (MET/week), RPE pre, RPE 10 min, RPE 15 min, RPE 20 min, and RPE post.

A χ^2^ test was used to compare gender among groups. Tukey post hoc test was also performed to find differences between them. Furthermore, a repeated-measure ANOVA (F) was used to analyze cortisol levels among groups (HC × MDD × AD) and within moments (preexercise and postexercise). The degree of freedom considered was Greenhouse–Geisser correction. Spearman correlation was performed to investigate correlation between delta cortisol and RPE pre, RPE post, HR pre, HR post, and IPAQ (MET/week). Linear regression was used to investigate the association among variables that showed difference in baseline condition (e.g., gender and hours of sleep) with delta cortisol.

To determine the magnitude of the difference in cortisol levels between pre- and post-values in HC, MDD, and AD groups, the effect size (ES) analyses were used. The results were interpreted according to Cohen's^27^ (trivial: d≤0.2; small: d=0.2–0.49; moderate d=0.5–0.79; and large: d≥0.8). Analyses were performed using SPSS^®^ software version 26.0 (IBM Corp., New York, USA), and figure was performed using GraphPad Prism^®^ software version 5.01. The p-value <0.05 was the threshold for statistical significance.

## RESULTS

Sample characteristics in baseline are presented in Table 1, showing that the three groups did not differ in age (F=2.208; p=0.129), years of education (F=2.311; p=0.118), VO_2max_ (F=2.435; p=0.106), MET_max_ (F=2.435; p=0.106), BMI (F=1.168; p=0.326), IPAQ (χ^2^=0.870; p=0.647), and IPAQ (MET/week) (χ^2^=1.275; p=0.528), while a significant difference was observed in gender (χ^2^=13.861; p≤0.001; MDD=HC<AD) and hours of sleep in the day before acute exercise (F=7.266; p=0.003; AD=MDD<HC). As expected, patients with AD showed a reduced global cognitive status compared to MDD and HC groups (χ^2^=18.392; p≤0.001; MDD=HC>AD). Moreover, MDD group presented higher depression symptoms than AD and HC groups (χ^2^=9.208; p=0.010; MDD=HC<AD).

**Table 1:** Descriptive characteristics of the sample in baseline.

	HC (n=10)	MDD (n=8)	AD (n=13)	p-value	Post hoc
Female*	10 (100.0)	7 (87.5)	4 (30.77)	≤0.001*	MDD=HC<AD
Age (years)	72.30±9.24	73.37±9.45	79.77±9.03	0.129	
Education (years)	16.30±7.53	11.87±4.19	11.23±5.28	0.118	
MMSE (score)*	29.00 (2.00)	29.00 (3.25)	20.00 (7.00)	≤0.001*	MDD=HC>AD
GDS (score)*	1.00 (2.25)	5.50 (10.25)	1.00 (4.50)	0.010*	MDD=HC<AD
BMI (kg/m^2^)	25.10±1.97	27.39±4.07	24.90±4.66	0.326	
VO_2max_ (mL/kg/min)	23.94±5.92	23.94±5.16	18.78±7.38	0.106	
IPAQ classification	4.00 (2.00)	2.00 (2.00)	3.00 (1.25)	0.647	
IPAQ (MET/week)	876.00 (4138.00)	495.00 (557.00)	871.50 (852.63)	0.528	
MET (mL/kg/min)	6.84±1.69	6.84±1.47	5.36±2.11	0.106	
Sleep (hours)*	7.00±1.05	6.50±1.19	8.54±1.50	0.003*	AD=MDD<HC

MMSE: Mini-Mental State Examination; GDS: Geriatric Depression Scale; HR: heart rate; BMI: body mass index; SD: standard deviation; MET: metabolic equivalent; VO_2max_: maximal oxygen consumption; IPAQ: International Physical Activity Questionnaire; HC: healthy control; MDD: major depressive disorder; AD: Alzheimer's disease.

* Significant difference among groups. Data expressed in mean±SD or median (interquartile range) or n (%).

Table 2 demonstrates values of RPE and HR pre-, during, and postexercise speed and slope of the treadmill in acute stressor test. However, some data were missing in RPE (five in HC and three in MDD groups) and HR (five in HC and two in MDD groups). There was statistical difference in RPE pre (χ^2^=6.266; p=0.044; MDD=HC<AD). There were nonsignificant difference observed for the other variables: RPE 10 min (χ^2^=5.358; p=0.069), RPE post (χ^2^=5.539; p=0.063), RPE 15 min (χ^2^=4.299; p=0.117), RPE 20 min (χ^2^=4.065; p=0.131), delta RPE (χ^2^=1.860; p=0.395), speed of treadmill (F=1.920, p=0.169), HR pre (F=0.273; p=0.764), HR 10 min (F=0.203; p=0.818), HR 15 min (F=0.122; p=0.886), HR 20 min (F=0.196; p=0.823), HR post (F=1.580; p=0.229), and delta HR (F=1.225; p=0.313). One-way ANOVA showed significant difference in treadmill slope during exercise (F=4.941; p=0.016; AD=HC<MDD), with significant difference between AD and HC groups (p=0.012) reaching the same exercise intensity stipulated in 70% of VO_2max_ during stressor test.

**Table 2:** Characteristics of the sample during acute exercise and changes (post–pre) with physical stressor.

	HC (n=10)	MDD (n=8)	AD (n=13)	p-value	Post hoc
RPE pre (score)	11.40±0.55	8.75±2.19	9.31±2.29	0.044*	MDD=HC<AD
RPE 10 min	12.80±0.45	6.87±5.79	12.00±1.96	0.069	
RPE 15 min	12.80±0.45	7.37±6.14	13.08±1.55	0.117	
RPE 20 min	13.20±0.45	7.62±6.32	13.61±2.02	0.131	
RPE post (score)	13.40±1.14	10.87±2.10	12.77±2.31	0.063	
ΔRPE (score)	2.00±1.00	2.12±2.69	3.46±2.93	0.395	
HR pre (score)	78.60±13.72	78.50±9.41	75.38±10.62	0.764	
HR 10 min	106.00±7.61	75.50±47.61	104.08±16.85	0.818	
HR 15 min	110.80±8.64	79.37±50.03	108.61±20.23	0.886	
HR 20 min	112.20±10.69	79.37±50.06	110.00±20.88	0.823	
HR post (score)	109.00±4.47	82.25±34.49	97.92±18.54	0.229	
ΔHR (score)	30.40±15.42	15.00±14.19	22.54±14.43	0.313	
Speed (km/h)	4.68±0.49	4.84±0.71	4.08±1.11	0.169	
Slope (%)*	7.70±4.79	4.25±1.83	2.85±1.62	0.016*	AD=HC<MDD

Δ : post (after exercise) − pre (before exercise);

HR: heart rate; HC: healthy control; MDD: major depressive disorder; AD: Alzheimer's disease; RPE: rating perceived exertion;

* Significant difference; SD: standard deviation. Data are expressed in mean±SD.

**Figure 1:**
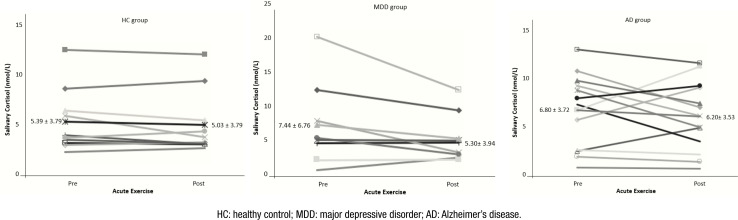
Effect of acute stressor (physical exercise) in cortisol levels in older individuals: healthy control group (A), major depressive disorder group (B), and Alzheimer's disease group (C). Data are presented in mean±standard deviation.

The repeated-measure ANOVA revealed no interaction for cortisol on the moment×group [F(2.000, 28.000)=1.285; p=0.293] and no effect for group (F=0.323; p=0.727). However, there was a significant effect for moment [F(1.000, 28.000)=4.930; p=0.035], with a decreased cortisol levels in postexercise for all groups (Figure 1). Gender as a covariate confirmed our outcomes and showed a trend to reduction in these levels (F=3.949; p=0.057). The comparison of delta cortisol among groups also showed nonsignificant difference (F=1.285; p=0.293). However, a reduction on cortisol levels of 6.5% for HC group, 8.8% for AD group, and 28.76% for MDD group was observed.

ES analysis revealed a small ES for patients with MDD (d=0.402) and trivial for patients with AD (d=0.166) and HC group (d=0.090). As expected, there was a positive correlation between HR pre and RPE pre (r=0.478; p=0.013) and between HR post and RPE post (r=0.478; p=0.016). Besides, in linear regression, nonsignificant results were observed for delta cortisol and gender (R^2^=0.088; p=0.106) and for delta cortisol and hours of sleep (R^2^=0.012; p=0.550).

## DISCUSSION

The main objective of this study was to investigate the cortisol reactivity to a single session of PE in MDD, AD, and HC elderly individuals. We hypothesized that cortisol levels would increase after acute PE in all groups, with results more pronounced for patients with MDD and AD. Notwithstanding, a significant cortisol reduction for all groups was observed.

The association between HPA axis dysfunction and MDD has been investigated over the six decades^28^; since then, both hypocortisolism and hypercortisolism at rest were found, which can impact the acute reactivity to a stressor agent. A previous meta-analysis^15^, with 43 articles in which 39 used pharmacological stressors and 6 used psychological tasks, concluded that aging increased the cortisol response to challenge. It was speculated that the lack of negative feedback resulting in higher cortisol levels was due to an impairment in brain structures, such as the hippocampus and prefrontal cortex generally observed in MDD^29^ and AD^30^. However, this explanation does not take into account some specific characteristics of these diseases in older individuals. For example, MDD older individuals are less likely to endorse affective symptoms and more likely to present cognitive changes, somatic symptoms, and loss of interest than are depressed young adults^31^.

MDD individuals seem to be more influenced by lifelong stress, which may be related to the exhaustion phase of stress-causing significant changes in the HPA axis^32^. A previous study demonstrated that lower evening cortisol levels may predict poorer course in elderly with MDD^33^. It is important to take into consideration the medication used by this population, because psychoactive medication was generally associated with lower cortisol levels^34^. Other study found a diminished ability to respond to the stress of awakening among depressed older persons^35^. In a longitudinal study, it was observed that a low mean salivary cortisol concentration and a small difference between morning and evening cortisol concentration may be risk factors of depression^36^. Moreover, apathy, fatigue, and exhaustion^37^ are very common symptoms in MDD and AD older individuals and are associated with hyporeactivity of HPA axis and consequent decrease in cortisol levels.

There are also other mechanisms related to aging. MDD and AD older individuals present more enhanced susceptibility to inflammaging^8^, which can affect the function of the glucocorticoid receptor (GR) and lead to hypercortisolism^38^ and consequently a worse memory^39^. Although a previous meta-analysis has observed hypercortisolism in AD in the mornings^12^, it is possible to hypothesize that individuals with more inflammaging and GR resistance can show reduced cortisol reactivity. It is also expected that AD individuals may encounter difficulties or even be incapable of evaluating the potential threat of a given stress event^40^. In contrast, a recent study showed that higher salivary cortisol reactivity to acute psychosocial stress test in healthy individuals aged 50 years or above was a hallmark of developing cognitive impairment 5 years later^17^, probably because senescence was turned to senility when occur inflammation and disorders come.

In addition to these speculations regarding MDD and AD characteristics, it is important to consider the type of stressor chosen. Physical stressor was less explored and previous studies have investigated adult populations with MDD and found nonsignificant blood cortisol changes after bicycle ergometer till exhaustion^41^ and a small increase of blood cortisol after bicycle ergometer till exhaustion^42^. A previous study with salivary sample observed cortisol reduction after bicycle ergometer with moderate intensity, which corroborates our results^43^.

In this study, cortisol reduction showed a small ES for all groups, with the highest effect for MDD compared to the other groups. Perhaps, these individuals had been more influenced by lifelong stress, which could be related to the exhaustion phase of stress-causing significant changes in the HPA axis^44^. Besides, other variables that could influence in results were not evaluated in this study, such as early life experience, chronic fatigue, social support, personality factors, chronic stress, and burnout^45-47^. We also have to point out the effect of acute stress using a rolling treadmill, because some individuals were extremely adapted to this type of stressor and others may experience some anxiety or afraid to be evaluated and anticipatory cortisol response must be considered. Meantime, this type of exercise is safe and adequate to use in this population.

### Strengths and limitations of the study and clinical relevance

This study has some limitations. First, we cannot generalize for all patients with AD and MDD because of the heterogeneity and individual characteristics (gender, hours of sleep, physical performance, and BMI) of the small sample. Another limitation was that we only took two moments to observe this reactivity, and we know that it is necessary and important to observe the curve of this reactivity with more moments before and especially 15, 30, 45, and 60 min after exercise.

Notwithstanding, it is important to highlight that this is the first study, to the best of our knowledge, to evaluate cortisol reactivity of a physical stressor in MDD and AD individuals. However, there is much to investigate. It is desirable to study the elderly population with neuropsychiatric disorders to better understand how these individuals may cope with stressor agent and also contribute to elucidate the physiopathology of such disorders regarding to the stress, while it is unavoidable to observe the heterogeneity of the subjects considering that it is inherent in this type of study.

Thus, the relationship between acute stress, healthy elderly, and, even more, those with neuropsychiatric disorders is still under debate and more research is required in future. This type of study has a pivotal relevance to clinical practice because their findings could provide adequate support assistance, care, and information to multidisciplinary professionals who follow up patients with AD and MDD. These professionals can offer treatment to prevent or minimize consequence and deterioration on memory and mood in these patients, improving quality of life. A future study with a larger and more balanced sample needs to be investigated, managing the influence of these variables in the outcome and considering that there is a cortisol variability within individuals of the same group.

Older individuals, regardless of the presence of mental disorder, show a decrease in salivary cortisol after a physical stressor. Nevertheless, this response seems to be more evident in MDD, which can be associated with chronic stress, HPA dysregulation, and a consequent inability to cope with stressor agents.
